# Development and internal validation of a predictive risk model for anxiety after completion of treatment for early stage breast cancer

**DOI:** 10.1186/s41687-020-00267-w

**Published:** 2020-12-04

**Authors:** Jenny Harris, Edward Purssell, Victoria Cornelius, Emma Ream, Anne Jones, Jo Armes

**Affiliations:** 1 School of Health Sciences, Faculty of Health and Medical Sciences, University of Surrey, Kate Granger Building, Priestley Road, Guildford, Surrey GU2 7YH UK; 2 School of Health Sciences, City, University of London, London, UK; 3 Imperial Clinical Trials Unit (ICTU), School of Public Health, Faculty of Medicine, Imperial College London, London, UK; 4 Florence Nightingale Faculty of Nursing, Midwifery and Palliative Care, King’s College London, London, UK

**Keywords:** Anxiety, Patient reported outcomes, Breast cancer, Predictive risk models, Cancer survivors, Supportive care

## Abstract

**Objective:**

To develop a predictive risk model (PRM) for patient-reported anxiety after treatment completion for early stage breast cancer suitable for use in practice and underpinned by advances in data science and risk prediction.

**Methods:**

Secondary analysis of a prospective survey of > 800 women at the end of treatment and again 6 months later using patient reported outcome (PRO) the hospital anxiety and depression scale-anxiety (HADS-A) and > 20 candidate predictors. Multiple imputation using chained equations (for missing data) and least absolute shrinkage and selection operator (LASSO) were used to select predictors. Final multivariable linear model performance was assessed (R^2^) and bootstrapped for internal validation.

**Results:**

Five predictors of anxiety selected by LASSO were HADS-A (Beta 0.73; 95% CI 0.681, 0.785); HAD-depression (Beta 0.095; 95% CI 0.020, 0.182) and having caring responsibilities (Beta 0.488; 95% CI 0.084, 0.866) increased risk, whereas being older (Beta − 0.010; 95% CI -0.028, 0.004) and owning a home (Beta 0.432; 95% CI -0.954, 0.078) reduced the risk. The final model explained 60% of variance and bias was low (− 0.006 to 0.002).

**Conclusions:**

Different modelling approaches are needed to predict rather than explain patient reported outcomes. We developed a parsimonious and pragmatic PRM. External validation is required prior to translation to digital tool and evaluation of clinical implementation. The routine use of PROs and data driven PRM in practice provides a new opportunity to target supportive care and specialist interventions for cancer patients.

**Supplementary Information:**

The online version contains supplementary material available at 10.1186/s41687-020-00267-w.

## Introduction

Symptoms of anxiety are frequently reported in response to breast cancer diagnosis and treatment [1]. Around 18–33% of women will experience anxiety following diagnosis of cancer [2, 3] and this may persist in 25% of women living with and beyond breast cancer (hereafter referred to as breast cancer survivors, BCS) two to four years after diagnosis, and in 15% after five [4]. Whilst internationally the importance of risk assessment for follow-up care is increasingly recognized [5, 6], there has been few attempts to identify those likely to experience late emotional and psychological effects amongst the growing number of cancer survivors and little application of modern approaches to data science and predictive risk modelling [7, 8]. In many countries follow-up care has shifted away from specialist cancer settings towards self-care and community-based services for BCS at low risk of cancer recurrence and late effects (physical and psychological) [7]. Given these changes, one approach to ensure psychosocial distress is assessed and addressed would be to use patient reported outcomes (PRO) alongside routinely collected data during cancer treatment to help identify those at increased risk of anxiety, and related conditions, long-term to further tailor supportive care services.

Predictive risk models (PRMs) aim to predict the risk of having or developing an outcome based on multiple variables [9]. To date studies using best practice approaches to PRM development have primarily focused on developing models for clinical outcomes [10]. PRMs have been developed and applied to inform screening, care and treatment recommendation in areas as diverse as organ transplantation [11], cancer [12, 13] and cardiovascular disease [14]. Development of a PRM of anxiety in BCS could be used to enhance follow-up care through heightened clinical awareness [10] [7]. However, although cancer policy identifies the need to offer extra support for high risk conditions such as anxiety [15, 16] as yet validated PRM stratification tools are lacking [7] and so are not routinely used.

Factors predictive of anxiety after completion of breast cancer treatment remain under-researched, use cross-sectional data [8] and are inadequately assessed [15], with little application of best practice guidance in the modelling process [17] [9, 18]. Traditional approaches to regression modelling focus on explaining phenomena rather than the ability to generate robust predictions [9, 18, 19]. This is common practice in psychosocial research where models are typically developed based on stepwise methods or univariate screening (typically including a variable in a model if it reaches a certain threshold of probability) [19]. Whilst such approaches attempt to reduce bias to obtain representations of the underlying data, this can lead to overfitting of the model to the data and reduce predictive performance in new data [20, 21]. If the goal is developing predictive risk models, statistical methods are required that minimize the combination of bias and estimation variance, occasionally sacrificing accuracy for improved precision and utility [9, 19]. A robust and useable PRM is generalizable, not overfitted to the data, and limits the number of variables that are required to be collected in routine practice [9, 19]. Applying robust methods to develop data driven and powerful PRM for psycho-oncology is paramount if we are to target on-going assessment and support in survivorship to those at greatest risk.

This study aimed to develop a predictive risk model, using principles of statistical learning to ensure robustness, [17] to determine the probability of experiencing anxiety in women with early stage breast cancer, to estimate the model’s predictive performance and undertake internal validation.

## Methods

### Study design

Secondary data analysis of a longitudinal cohort, the Supportive Care Needs Study (SCNS) of people receiving treatment with curative intent. This secondary analysis focused on women diagnosed with breast cancer only [22]. Ethical approvals were obtained as part of the original study.

### Participants and procedures

Sixty-six cancer centres in England participated in the SCNS during 2005. Eligible patients were: ≥18 years; able to read English; undergoing curative treatment; women diagnosed with breast cancer. Nurses recruited consecutive eligible patients. Participants self-completed a postal survey after their final treatment (T0), and 6 months later (T1), with non-response reminders. Previously published work suggests the sample was representative of cancer centres in the UK [22].

### Outcome measure

The PRO was Hospital Anxiety and Depression Scale (HADS)-Anxiety (HADS-A) [23] total sub-scale score 6-months after treatment completion (T1). HADS-A includes seven items to compute a total anxiety score (range 0–21). In clinical practice HADS-A is widely used as a screening tool and is both valid and reliable for identifying symptoms of anxiety in cancer and other clinical populations [24, 25].

### Potential predictors

Selection of predictors for inclusion in the model was informed by results from a systematic review [8], written feedback from people with cancer contacted through a patient advocacy group and an expert advisory group. They provided feedback on potential ease of use in clinical settings (e.g. are data routinely collected or easily accessible?) and patient views (e.g. is it acceptable?), an important consideration if a PRM is to be implemented in routine clinical practice [10]. An overview of candidate predictors considered in the model is provided in Table 1. These included socio-demographic (age, marital status, caring responsibilities, employment status, highest educational level, car ownership, housing tenure, self-reported financial strain), psychological (prior poor mental health as indicated by T0 HADS) and clinical variables (comorbidities, disability, type of cancer treatment, self-report of feeling sick, fatigue or pain). Several pre-determined interactions based on previous evidence were considered including symptoms (pain, fatigue and depression) [26] and economic factors (economic status, homeownership and financial strain) [27].

**Table 1:** Sample characteristics

Candidate predictors	N (%)^a^
**Socio-demographic**	
**Age:**	
19–51 years	250 (31.1)
52–59 years	192 (23.9)
60–65 years	150 (19.7)
66–71 years	109 (13.7)
72+ years	102 (12.7)
Mean age	58.0 (SD 11.5) (range 27–88)
*Missing*	*13 (1.6)*
**Marital status**	
Married or living with partner	590 (72.5)
Widowed	92 (11.3)
Divorced / Separated	84 (10.3)
Single	48 (5.9)
*Missing*	*2 (2.3)*
**White British ethnicity**	761 (93.6)
*Missing*	*3 (0.4)*
**Lives alone**	140 (17.4)
*Missing*	*11 (1.4)*
**Housing tenure**	
Owner-occupier	694 (85.3)
Renting	104 (12.8)
Other	16 (2.0)
*Missing*	*2 (0.3)*
**Have any caring responsibilities**	227 (28.5)
Missing	*19 (2.3)*
**Highest level of qualification**	
No formal qualification	286 (35.6)
A level or equivalent	97 (12.1)
GCSE/O Level	242 (30.1)
Degree/higher degree	178 (5.1)
*Missing*	*13 (1.6)*
**Employment status**	
Working	268 (33.2)
On leave	130 (16.1)
Retired	329 (40.7)
Not working	81 (9.5)
*Missing*	*8 (0.3)*
**Use of car or van**	709 (87.0)
*Missing*	*19 (2.3)*
**Clinical**	
**Mean HADS-A (baseline)**	6.5 (SD 4.2, median 6.0, IQR 6.0)
*Missing*	*14 (1.4)*
**Mean HADS-D (baseline)**	3.5 (SD 3.2, median 3.0, IQR 4.0)
*Missing*	*4 (0.8)*
**Moderate or severe need: feeling unwell**	64 (8)
*Missing*	*15 (1.8)*
**Moderate or severe need: lack of energy/fatigue**	164 (20.3)
*Missing*	*8 (1.0)*
**Moderate or severe need: feeling pain**	70 (8.7)
*Missing*	*14 (1.7)*
**Longstanding comorbid illness**	317 (39.2)
*Missing*	*7 (0.9)*
**Cancer-related treatments**	
**Chemotherapy**	350 (64.6)
*Missing*	*274 (33.6)*
**Radiotherapy**	766 (96.6)
*Missing*	*23 (2.8)*
**Hormone therapy**	537 (69.3)
*Missing*	*41 (5.0)*
**Outcome**	
**Mean HADS-A** ^b^	6.8 (SD 4.4)
*Missing*	150 (18.4)

^a^ Candidate predictor counts and percentages are for valid responses, except for missing data which represents overall figure

^b^ Values for MI data. Complete case values were mean 6.7 (SD 4.3, median 6.0, IQR 7.0)

### Statistical analysis

Continuous predictors including age, HADS-A and HADS-Depression (HADS-D) were used to retain maximum predictive information [28] and for later translation of the tool into different clinical practices where appropriate risk grouping can be determined [29]. No author guidance exists for handling missing values in the HADS questionnaire, so a pragmatic approach was adopted in line with recent evidence [30]. If an item was missing for HADS sub-scale all other scores for that participant were used to impute the mean value for the missing item [30]. If > 2 items were missing the whole HADS sub-scale was treated as missing. We examined associations with missingness using a series of logistic regressions to inform selection of variables included in the imputation models [31].

Missing data were handled using multiple imputation using chained equations (MICE) [32] to impute 50 datasets [32, 33] with predictive mean matching for continuous data (supplement 1). HADS scores had an approximately Gaussian distribution and extensive assessment was undertaken of model specifications for both the imputation and main analysis models. For example, collinearity was explored using variance inflation factors, margins, margins plots and contrasts (data available upon request).

#### Selection of candidate predictors

Stakeholder involvement highlighted that the potential list of > 20 predictors would be unwieldly to use in clinical practice and unacceptable to patients, so this needed to be reduced. Traditionally analysts used univariate screening or stepwise regressions to achieve this, where inclusion of variables in a model is overdependent on null hypothesis significance testing and based on pre-determined criteria (e.g. *p* values) [19]. However, it is now widely accepted that this should be avoided because it can lead to poor estimation, is biased in selection and can result in model overfitting [19, 34]. These weaknesses limit the generalizability of the resulting regression model when intended for prediction purposes [21, 35].

Modern regularization techniques provide a powerful alternative to overcome these problems [21]. In this secondary analysis Least Absolute Shrinkage and Selection Operator (LASSO) [36] was used as it produces parsimonious models and can help to minimize prediction error and overfitting by reducing the regression coefficients. This is achieved by introducing a penalty term [19, 21, 37] whereby the penalty term is equal to the sum of the absolute coefficient, meaning all coefficients are shrunk and some reduce to zero [38]. Those reduced to zero are effectively removed from the model, making it a useful tool for developing parsimonious PRM to be used in clinical practice. To put simply, LASSO introduces some constraint which prevents the coefficients estimates having an inappropriately large magnitude [39].

LASSO was implemented for each imputed dataset, with predictors selected if they were included in ≥50% of the models [32, 40] or if they were a known predictor (age, anxiety and depression) [8]. LASSO was performed for all candidate predictors and again separately for all candidate predictors plus interactions. The final model’s predictive performance was internally validated using Bootstrapping based on MI dataset. By generating a new sample of data from the original sample this provides estimates to account for model overfitting or uncertainty in the entire model development process [17]. Bootstrap distribution for the predictors across 1000 results was compared to those of the original model. Model discrimination was assessed by the explained variance (R^2^) [19]. All analyses followed recommendations for multivariable predictive research [17, 35, 41, 42]. To compare with standard approaches to model development we also built a model using univariate screening on the complete-case data, whereby a candidate predictor was included in the multivariable model if unadjusted *p* ≤ 0.10 [19]. Data analysis was performed using Stata (version 15) [43].

## Results

**Fig. 1:**
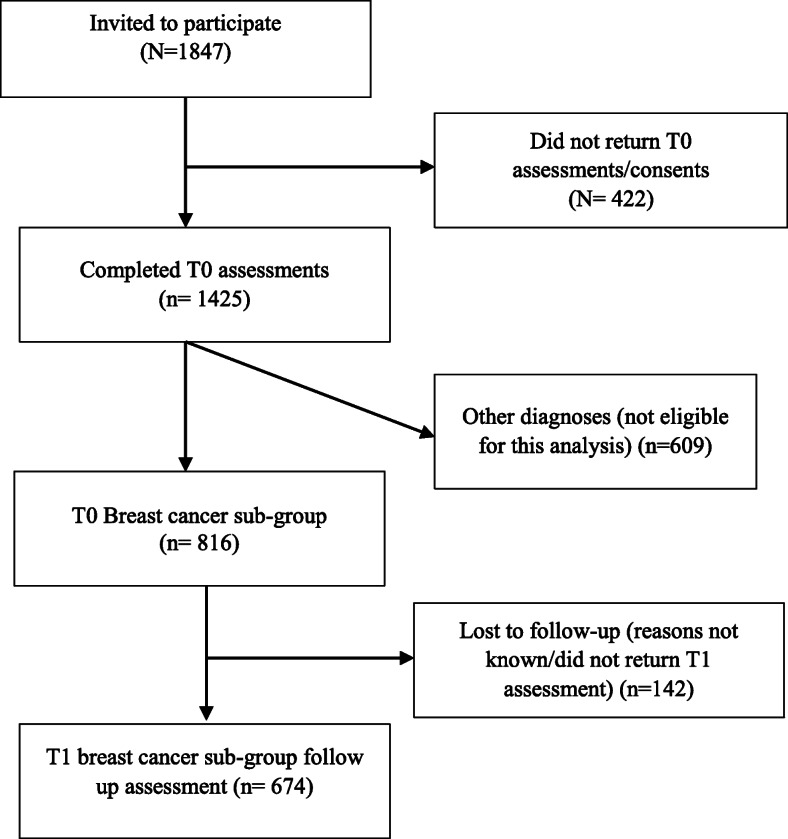
Flow chart of participants’ study inclusion. T0, baseline (at the end of treatment); T1, 6 months after baseline

There were 1847 people who agreed to participate in the original study and 1425 returned the initial survey (79%). The sample included 816 women with breast cancer who were included in this study, of whom 674 returned surveys at T1 (Fig. 1).

Participant characteristics are presented in Table 1. Mean age was 58 years (SD 11.51), most were married or living with a partner (72.5%) and White British (93.6%). Mean anxiety score was 6.5 (SD 4.2) at T0 and 6.8 (SD 4.34) at T1.

### Predictive risk model of anxiety

**Fig. 2:**
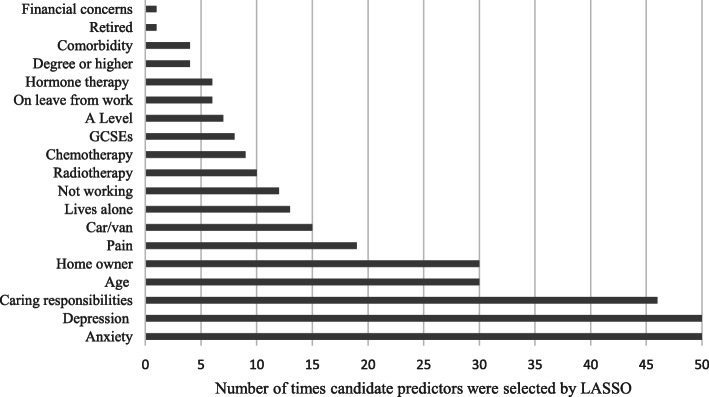
Number of times candidate predictors were selected by LASSO in MI datasets (m = 50)

From the 20 candidate predictors, five were selected by LASSO in > 50% of the MI datasets (Fig. 2, supplement 2) [40]. When examining the LASSO including additional interactions, two additional predictors were selected (financial strain and working status; working status and homeownership), however, inclusion of these did not improve model performance and so the parsimonious five predictor model was chosen (not presented but available on request).

The final model predicted that T1 anxiety scores are higher with increasing scores on T0 HADS-A, HADS-D and having caring responsibilities but decrease with older age and being a homeowner (Table 2). The final model explained 60% variance in the data. Bootstrap validation results suggest the level of bias for the final model was low (− 0.006 to 0.002%, Table 2), indicating stable accuracy of the estimate between the original and bootstrap samples. In comparison the univariate screening approach resulted in the inclusion of ten predictors (age, living alone, caring responsibilities, homeowner, financial need, employment status, lack of energy/fatigue, feeling unwell, HADS-A, HADS-D) and explained 59% of variance in the data (supplement 3).

**Table 2:** Prediction model estimates and bootstrap estimates

Predictor	MI estimate			MI bootstrap estimate				
	B	SE	95% CI	B_**b**_	SE_**b**_	Z	Bias	B 95% CI
**HADS-A**	0.734	0.034	0.67,0.80	0.734	0.027	27.12^a^	0.000	0.68, 0.79
**HADS-D**	0.094	0.043	0.01, 0.18	0.095	0.041	2.28^b^	0.001	0.02, 0.18
**Age**	−0.011	0.011	−0.03, 0.01	−0.010	0.008	−1.30	0.001	− 0.03, 0.01
**Caring responsibility**	0.485	0.274	−0.05, 1.03	0.488	0.200	2.43^a^	0.002	0.08, 0.87
**Homeowner**	−0.426	0.326	−1.07, 0.22	−0.432	0.263	−1.62	−0.006	−0.95, 0.08
**Constant**	2.515	0.793	0.95, 4.08	2.475	0.587	4.28^b^	−0.040	1.39, 3.67

MI estimate: B = MI observed coefficient, SE B = standard error of B, 95% CI (confidence intervals)

MI Bootstrap estimate: B_b_ = MI bootstrap estimates of coefficient, SE_b_ = standard error of B_b,_ z = bootstrap estimate divided by the standard error, bias = bias for the parameter estimate, B 95% CI = bias corrected 95% CI

All estimates are based on MI data (M = 50) and Bootstrap distribution across 1000 results (10,000 random samples with replacement) ^a^*p* < 0.05 ^b^ *p* < =0.001

Simple 6-month predicted anxiety = 2.5 + (HADS-A score × 0.7) + (HADS-D score × 0.1) + (age x − 0.1) + 0.4(if carer) + − 0.4(if homeowner)

## Discussion

This study developed a parsimonious PRM for anxiety 6-months following breast cancer treatment completion, using LASSO regression to identify the most salient predictors. Few studies have attempted to develop multivariable predictive risk models for patient reported anxiety breast cancer treatment [8]. Anxiety at the end of treatment was an important predictor of anxiety 6-months later, a finding that is supported by previous research [8] and policy [16]. In this study we used HADS-A, but other screening tools can be used in practice with similar performance [35, 36]. Good screening tools are typically quick to administer, for example HADS takes 2–5 min to complete [37], and advances in digital data collection mean this can be linked with other important predictive data in real-time.

In psycho-oncology existing models of outcomes tend to be explanatory, attempting to elucidate causal mechanisms. Here the goal was prediction and so we used a data driven approach. We intended the model to be practical; for it to be useful in clinical practice it needed to include variables that are either routinely collected or required limited additional information to be collected. In contrast, the model built using traditional univariate screening resulted in twice the number of predictors being including in the model and would require greater clinical effort and resource to collect.

We found LASSO regression, increasingly used in machine learning and precision medicine but rare in psycho-oncology, was a helpful method to identify predictors and multiple imputation enabled us to fully utilize the dataset. We would encourage wider use of robust statistical techniques like these for data driven model development for psychological outcomes in medicine. LASSO allowed us to identify the most relevant predictors of anxiety at follow-up and develop a parsimonious PRM.

An important aspect of predictive rather than explanatory modelling is that variables included in risk models will not necessarily be individually statistically significant [21]. However, they may still be important to include to fine-tune performance as the aim is not hypothesis testing [19]. Thus, they can have clinical significance even in the absence of statistical significance. Younger age was identified as a possible predictor of anxiety after treatment finished in previous research [8]. Although only weakly associated here, age was still selected by the LASSO procedure. Social risk factors, although generally thought to be predictive of anxiety for people with other chronic health conditions [44], have not been consistently identified in BCS [8]; using LASSO the small but important effects of social factors helped us refine the model. Housing tenure and having caring responsibilities have not been identified as predictors in previous research and it may be that specific predictors are more relevant to certain social, economic and cultural contexts. For example, in the UK homeownership may serve as a proxy for social economic status but may not be an important predictor in locations where renting is more widespread. Further, the definition of caring responsibility was quite broad as it could include any caring for an adult or child. Future research is needed to determine if it is caring per se, or whether it is responsibility for an adult, child or both that is important. There is some evidence that the risk of anxiety is greater during chemotherapy treatment than other treatment modalities [45]. However, in line with previous research [8], chemotherapy did not predict anxiety after treatment completion. This finding is an important message for patients and clinicians.

### Limitations

This study was limited to secondary analysis of a pre-existing data. Longitudinal research is expensive and funding scarce, so it is generally recognized that there is a need to use existing datasets for predictive modelling [46] and that data should ideally be observational and comprehensive, as with the current study. Nonetheless, there may be other important variables (lifestyle factors, newer treatments and adverse-events) not measured in primary research that may impact on anxiety. Indeed, these could be important candidate predictors to consider in future research to account for the variance unexplained in the model. However, this study completes the first stage in a development process that we will build upon in future model validation studies. Furthermore, a third of chemotherapy treatment data was missing which may present some reporting bias although we attempted to mitigate this using MICE. It was an advantage that this study used data that is widely collected and a tool that is well understood by clinicians and researchers.

The original research was conducted with a sample of women with early stage disease and good oncological prognosis. Results may be different for women with later stage disease, many of whom will live for many years with incurable disease. Further research is needed to determine predictors in this group. The study sample was limited to include only women who could read English and future research is needed to examine its predictive performance in across the diverse communities in the UK and its utility in international clinical contexts. Indeed, these issues have been noted as limitations of well-known PRM in cancer such as ‘Adjuvant’ and MammaPrint’ [47].

To maintain predictive sensitivity the model was developed using a continuous outcome making immediate clinical interpretation less straightforward than if a model with a dichotomous outcome had been developed. However, this was deliberate, and we feel necessary, given the early stage of evidence in this area and to make the model more sensitive. Further, digital technologies allow greater ease of use and interpretation of such outcomes. Another limitation was the preliminary nature of this study and the PRM requires validation in an external sample with decision-curve analysis to determine the clinical impact of different thresholds [48]. Validation of this model in external samples will help us to identify individuals at low, moderate or high risk of anxiety after treatment completion, through calculation of risk scores/algorithms, ready for translation into a digital tool and evaluation of clinical implementation in future research. High quality longitudinal data from similar cohorts is expensive to collect and currently scarce, therefore we plan to undertake secondary analysis of trials using PROs to further refine and externally validate the model.

Future research is needed to determine appropriate cut-offs based on best practice regarding predictive accuracy and clinical utility [21], and whether standard HADS-A thresholds are appropriate for this population [24] and adaptions using different outcome measures. For example, by examining the utility of different screening tools where it might be possible to calibrate and adapt PRM depending on the preferred instrument already used in clinical setting. Long-term such tools may help health services plan their resourcing and provision of follow-up care according to patient characteristics, permitting stratified follow-up with different support options for different levels of risk.

A strength of this study was the modelling strategy, it followed recommendations to limit bias in the identification of predictors. For example, in many studies model building procedures such as variable selection is undertaken on complete-case datasets, even where MI has been used. Current guidelines do not support this approach as the results can be biased and lack power [32, 40].

## Conclusions

A myriad of individual predictors of anxiety for breast cancer survivors have been identified in previous research. It is impractical and unnecessary for these all to be collected and entered into a digital PRM in clinical practice by busy specialist cancer nurses or oncologists often responsible for referring to supportive or specialist psycho-oncology services. Further, previous research has not used sophisticated statistical learning approaches now recommended for developing PRM. This study developed a parsimonious PRM for anxiety after breast cancer treatment that, if further validated and refined, has the potential to be adapted to a digital tool to be used in clinical practice. At completion of treatment anxiety, depression and having caring responsibilities increased risk for anxiety, whereas being older and owning a home were protective. The methods presented here may provide a useful framework for others wanting to harness the power of data driven predictive models for psychological patient reported outcomes in clinical populations. PRMs present the opportunity to facilitate the use of routinely collected patient reported predictors and outcomes to enhance patient quality of life through stratified supportive care packages.

## Supplementary information

Additional file 1: **Supplement 1.** Summary of final imputation model specification. **Supplement 2.** Illustration of the LASSO estimates. **Supplement 3**. Estimates for model selection using univariate screening.

## Data Availability

The dataset analysed during the current study are not publicly available because this was a secondary analysis but are available via the corresponding author on reasonable request with permission from Dr. Jo Armes.
