# Supplementary material for: Development and internal validation of a predictive risk model for anxiety after completion of treatment for early stage breast cancer
Source: J Patient Rep Outcomes. 2020 Dec 4;4:103. doi: 10.1186/s41687-020-00267-w (PMC7718350; doi:10.1186/s41687-020-00267-w)
Supplement: Supplementary file 1 — Supplement 1. Summary of final imputation model specification. Supplement 2. Illustration of the LASSO estimates. Supplement 3. Estimates for model selection using univariate screening. [file 41687_2020_267_MOESM1_ESM.docx]

**Supplementary materials**

**Tables**

**Supplement 1 Summary of final imputation model specification**

| Multiple Imputation by Chained Equations (MICE) implemented in Stata 15 using commands mi impute chained | |
| --- | --- |
| **Variables** | **Type of imputation** |
| HADS-A T1  HADS-A T0  HADS-D T0  Age in years* | Predictive mean matching (pmm) using the 10 closest nearest neighbors |
| Regular use of a car or van  Lives alone*  Has caring responsibilities  Homeowner  Financial need*  Report feeling pain  Any comorbidities  Report lack of energy/fatigue  Report feeling unwell  Chemotherapy treatment  Radiotherapy treatment  Hormone therapy | Logistic regression (logit), entered as binary variable |
| Employment status | Multinomial regression (mlogit), entered as factor variable |
| Highest educational qualification | Ordered (proportional) regression (ologit), entered as factor variable |
| **Number of iterations (burn-in period):** 10 | |
| **Linear rule for missing data:** 44% of cases missing some data, at least 44 imputations | |
| **Fraction of missing data using quadratic rule:** 0.38 (95% CI 0.29,0.47), 46 imputations minimum | |
| **Final number of imputations:** 50 | |

*associated with missingness

**Supplement 2 Illustration of the LASSO estimates**

| **Candidate predictors** | | **LASSO selection** |
| --- | --- | --- |
|  | | **LASSO^+^** |
| **Age** | | -0.001 |
| **Lives alone** | | 0 |
| **Regular use of car or van** | | 0 |
| **Has caring responsibilities** | | 0.244 |
| **Homeowner** | | -0.008 |
| **Self-report financial need** | | 0 |
| **Employment status** | Working  On leave  Retired  Not working | 0 |
| **Highest educational qualification** | None  GSCE  A level  Degree | 0 |
| **Has any comorbidity** | | 0 |
| **Report feeling pain** | | 0 |
| **Report lack of energy/fatigue** | | 0 |
| **Report feeling unwell** | | 0 |
| **Had chemotherapy** | | 0 |
| **Had radiotherapy** | | 0 |
| **Had hormone therapy** | | 0 |
| \|  \| \| --- \|   **HADS-A T0** | | 0.739 |
| **HADS-D T0** | | 0.032 |
| **Constant** | | 1.93 |
| **R-squared** | | 0.60 |

+ LASSO: least absolute shrinkage selection operator. Example is from M=49. LASSO does not produce standard errors and so are not presented.

**Supplement 3: Estimates for model selection using univariate screening**

| **Predictor** | **Univariate screening estimate** | | | **Univariate screening bootstrap estimate** | | | | |
| --- | --- | --- | --- | --- | --- | --- | --- | --- |
|  | **B** | **SE** | **95% CI** | **B_b_** | **SE_b_** | **Z** | **Bias** | **B 95% CI** |
| **HADS-A** | 0.73 | 0.03 | 0.67, 0.80 | 0.74 | 0.03 | 22.52 | -0.001*** | 0.68, 0.81 |
| **HADS-D** | 0.13 | 0.05 | 0.04, 0.22 | 0.13 | 0.05 | 2.54 | 0.003* | 0.03, 0.24 |
| **Age** | -0.01 | 0.02 | -0.04, 0.02 | -0.01 | 0.02 | -0.55 | -0.001 | -0.03, 0.02 |
| **Caring responsibility** | 0.46 | 0.29 | -0.11,1.02 | 0.46 | 0.30 | 1.52 | 0.016 | -0.14, 1.05 |
| **Homeowner** | -0.48 | 0.35 | -0.21,1.17 | 0.48 | 0.38 | 1.26 | 0.021 | -0.26, 1.23 |
| **Lives alone**  (compared to lives with other) | 0.14 | 0.32 | -0.48,0.76 | 0.14 | 0.35 | 0.40 | -0.009 | -0.54, 0.82 |
| **Self-report financial need**  (compared to no financial need) | -0.14 | 0.39 | -0.91,0.63 | -0.14 | 0.42 | -0.33 | -0.013 | -0.96, 0.69 |
| **Employment status**  Working  On leave  Retired  Not working | -  -0.27  -0.06  0.12 | -  0.34  0.34  0.42 | -  -0.94,0.40;  -0.73, 0.62  -0.69, 0.95 | -  -0.27  -0.06  0.13 | -  0.37  0.35  0.44 | -  -0.72  -0.16  0.29 | -  -0.017  0.009  -0.004 | -  -0.95, 0.44  -0.81, 0.59  -0.68, 1.05 |
| **Report lack of energy/fatigue**  (compared to no problems with energy/fatigue) | -0.04 | 0.35 | -0.74,0.65 | -0.04 | 0.34 | -0.13 | -0.007 | -0.72, 0.63 |
| **Report feeling unwell**  (compared to no problems with feeling unwell) | -0.42 | 0.52 | -1.45,0.61 | -0.42 | 0.57 | -0.73 | -0.008 | -1.54, 0.70 |
| **Constant** | 1.40 | 0.93 | -0.47,3.27 | 1.40 | 0.89 | 1.57 | -0.002 | -0.35, 3.15 |
| Univariate screening estimate includes all candidate predictors associated with the outcome above or equal to the threshold of p = 0.10. B= observed coefficient, SE B = standard error of B, 95% CI (confidence intervals). Adjusted R squared was 0.59  Univariate screening bootstrap estimate: B_b_= bootstrap estimates of coefficient, SE_b_ = standard error of B_b,_ z= bootstrap estimate divided by the standard error, bias= bias for the parameter estimate, B 95% CI= bias corrected 95% CI.  All estimates are based on complete-case and Bootstrap distribution across 1000 results (10,000 random samples with replacement) *p<0.05 *** p<=0.001 | | | | | | | | |
